# Family members’ reflections upon a family-based inpatient treatment program for adolescent anorexia nervosa: a thematic analysis

**DOI:** 10.1186/s40337-020-00360-x

**Published:** 2021-01-06

**Authors:** Jan-Vegard Nilsen, Øyvind Rø, Inger Halvorsen, Hanne Weie Oddli, Trine Wiig Hage

**Affiliations:** 1 Department of Psychology, University of Oslo, Oslo, Norway; 2 Regional Department for Eating Disorders, Division of Mental Health and Addiction, Oslo University Hospital, Oslo, Norway; 3 Institute of Clinical Medicine, University of Oslo, Oslo, Norway

**Keywords:** Eating disorders, Anorexia nervosa, Family-based treatment, Inpatient treatment, Qualitative research, Higher levels of care, User perspectives

## Abstract

**Background:**

Family-based outpatient treatment is usually recommended as the treatment of choice when a child develops anorexia nervosa. However, some young persons will inevitably require higher levels of care. Qualitative research on family perspectives may help inform strategies to adapt family-based practices into intensified treatment settings. Our overarching aim was to investigate family members’ perspectives following a family-based inpatient treatment program for adolescent anorexia nervosa and to discuss clinical implications for treatment providers.

**Methods:**

A subsample of eight families taking part in a naturalistic outcome study at a specialized eating disorder unit participated in the study (8 patients, 14 parents, and 10 siblings). The thematic analyses were inductive, predominantly descriptive, and guided by a multi-perspective framework.

**Results:**

Five main themes were identified: 1: *Expectations and evaluation of needs. Entering treatment from different vantage points*, 2: *Interactions with peers during the admission as highly beneficial or problematic*, 3: *Perspectives on staff expertise and the eating disorder unit’s structure*, 4: *Influencing within family relationships in different ways*, and 5: *Being admitted is at best only half the job: reflections on leaving the eating disorder unit*.

**Conclusions:**

Our study offers insight into how former inpatients and their family members experienced an inpatient treatment program designed to align treatment with the central elements of an outpatient family-based treatment approach for adolescent anorexia nervosa. Overall, the findings support emerging research underlining the necessity of strengthening the family-based treatment approach within intensified treatment settings. Moreover, the results emphasized the need for more knowledge on how to optimize inpatient treatment as well as the importance of providing smooth transitions between care settings.

## Plain English summary

Inpatient treatment of anorexia nervosa has traditionally been individually based. At large, this has usually meant that the young person with anorexia nervosa has been separated from their family during a hospital admission, while parents and family-members have had the chance to visit for treatment meetings, support and therapy sessions. Inspired by the promising research on outpatient family-based treatment, a treatment model that put a lot of emphasis on supporting the parents and “the family as a whole” during treatment, some treatment centers around the globe has started to hospitalize parents and siblings together with the young person with anorexia nervosa. The present study offers insight into how family members have experienced taking part in such a family-based inpatient treatment program. The family members demonstrated considerable diversity in viewpoints. Without prescribing definitive answers, we believe the results have several important implications for treatment providers working within a family-based inpatient treatment approach.

## Background

Outpatient family-based treatment, either the well-known “Maudsley approach” [1] or manualized family-based treatment (FBT-AN) [2], is usually recommended when a young person develops anorexia nervosa (AN) [3]. Still, inpatient treatment is often required for child- and adolescent AN, both because of the complexity and symptom severity, but also due to the lack of accessible recommended outpatient treatments in many regions [4, 5]. Inpatient treatment is also frequently used when a young person with severe AN does not achieve adequate progress at an outpatient treatment facility [6, 7]. For some, a more intensive level of care is required even when the young person and their family receives highly specialized, evidence-based outpatient treatment, as no treatment is a panacea [8].

Inpatient treatment demands a lot of resources, including human and financial [4, 9]. Availability is typically limited, as most specialized treatment centers have strict admission criteria and offers only a few beds for a large catchment area [7, 10]. Inpatient care is recognized as a highly multifaceted and complex endeavor, and to date, no internationally agreed upon treatment guidelines for AN exist to guide clinicians on how to efficiently and effectively provide and manage inpatient care [4, 11]. Importantly, inpatient care has shown to have uncertain long-term effects, as many of the patients fail to maintain improvements achieved during admission [4, 12]. The latter is mirrored in the relative high relapse rates for this population-at-large, and underscores the importance of improving inpatient care as well as collaboration with the referral system to facilitate transitions [13].

Developing better ways to optimize the inpatient treatment setting for young persons with AN has been called for by recent ED studies [4, 11]. Due to promising evidence from the last two decades of development and research on outpatient family-based treatments, some treatment centers around the globe have begun to incorporate key tenets of outpatient family-based treatment into higher levels of care [6, 7, 10, 14, 15]. Most developments have been pioneered by highly specialized treatment centers aiming to align the core features embedded in evidence-based FBT within intensified treatment programs. This work aims to both optimize the provision of care during hospitalizations and importantly, to enhance the maintenance of effects following discharge [7, 10, 11, 14].

Research investigating the potential benefits of adapting family-based interventions at higher levels of care is emerging, yet remains scarce [7, 16–19]. A recent study from an Australian context investigated the effects of a brief admission prior to outpatient FBT and showed that admitting the family for an intensified two-week program offered the families an opportunity for relational strengthening and re-unification, thereby providing a stronger foundation for outpatient FBT [18]. Another study found that although outpatient FBT cannot be replicated at higher levels of care, treatment principles can be effectively adapted to a day treatment program [6, 16]. Our own research on outcome following admission to a family-based inpatient treatment program also found that enhancing the family-based focus during hospitalization is a promising approach for those who fail to respond to outpatient treatment [7].

With the current study, we aimed to extend our prior research on a family-based inpatient treatment approach for adolescent AN, which has to date focused upon outcome [7], treatment satisfaction [20], siblings’ experiences [21], and user experiences [19]. In the present study, we provided a multiperspectival approach to extend our knowledge beyond the single-position approach previously applied in our qualitative studies [19, 21, 22]. Specifically, the research question focused upon how to characterize the multiple user perspectives of parents, siblings and patients’ belonging to a single family following admission to a family-based inpatient treatment program at a specialized eating disorder unit (EDU) for adolescents with AN.

## Methods

### Research design

The study was a qualitative descriptive study and formed part of a larger naturalistic outcome study investigating different aspects related to a family-based inpatient program.

### Ethics

Ethics approval was obtained from the Regional Committee for Medical Research ethics, South East Norway [REK2014/2223]. All participants provided written consent to take part in this research. All names in the results section are pseudonyms.

### Participants and sampling procedure

Post-treatment perspectives from eight former inpatients and their family members were included (8 former patients, 14 parents, and 10 siblings). All participants took part in a family-based inpatient treatment program at a specialized EDU between 2008 and 2014. This subsample was purposively derived from the complete data set of thirty-seven inpatients participating in the main outcome study [7]. The eight families were selected because we had post-treatment interview data from patients, as well as their siblings and parents. This sampling choice mirrored our aim to investigate user perspectives from multiple positions within a single family and enable within and between family comparisons.

Mean age at admission was 15 years (range: 12–18) and mean age at follow up was 19 years (range: 16–21). Mean length of stay was 21.4 weeks (range: 8–58), including planned leaves as part of the treatment program. All patients had an admission diagnosis of AN. No statistically significant differences existed between the 8 participants and the 29 non-participating patients for the following variables: age at admission, duration of ED before admission, length of stay, weight and BMI percentile at admission and discharge, time elapsed between discharge and follow up and EDE-Q global score at follow up.

All patients had received previous outpatient treatment at specialized mental health services, as well as prior inpatient treatment at a medical and / or psychiatric unit. During the follow-up interviews, 5 of the former patients did not meet the criteria for any DSM-5 ED diagnosis. In seven of the eight families, the parents were married. Six of the parent interviews were conducted with both parents together; two of the interviews were conducted only with the mother. Siblings’ mean age at admission was 11 years (range: 6–16) and mean age at follow up was 15.4 years (range: 11.9–23). None of the siblings reported any previous treatment experiences beyond visitation during hospitalization of their sibling. Only one of the siblings had attended a family session at the local outpatient clinic.

Overall, our sampling strategy was guided by the acknowledgement that each and every participant inevitably represented themselves and their subjective opinions and perspectives, and further, that the themes generated through the qualitative analysis would be judged more or less as representative or relevant within different clinical contexts by the reader, an approach to generalization often recognized as a case-to-case transferability [23].

### Treatment setting

In 2008, the EDU changed the treatment program in order to provide a family-based inpatient treatment program. This restructuring was guided by a) the promising evidence-base from research on outpatient family-based therapy for AN, b) the wish to prevent separating the ill child from caregivers during hospitalizations and c) the specific legal situation in Norway, where children have a legal right to be accommodated by a parent during hospitalization. Since then, up to 5 families have been treated at the same time [7].

As outpatient FBT was originally a manualized version of the family-based treatment approach developed at the Maudsley hospital, the EDUs inpatient treatment program has undoubtedly been influenced by both sources. Although these two outpatient treatment models are today recognized by some differences, they share the common core features that, taken together, have been influential for treatment adaptations at the EDU. These features include charging parents with more responsibility during the admission (i.e., continually aiming to facilitate parental empowerment throughout treatment), externalizing the ED, together with aligning treatment with the non-blaming/non-etiological/non-authoritarian therapeutic stances embedded in both outpatient treatment models. Although the EDU has not adhered to a strict manualized FBT approach, the EDU has continually aimed to align treatment with the core features and therapeutic stances associated with outpatient FBT [2, 11, 24]. Generally, the treatment program corresponds to the first phase of outpatient FBT, as the main aim during admissions has been to enable AN symptom improvement. Contrary to standard outpatient FBT, however, the treatment team has had the final say on the meal plans during admissions. This decision-making process has commonly been done in close collaboration with the parents and only when viewed appropriate involved the patient, during the weekly treatment meetings (i.e., dependent on progress). During later phases of hospitalization, the focus has gradually shifted towards encouraging the adolescent to assume more responsibility for eating, with continued parental supervision. All families had weekly treatment meetings with a multidisciplinary team. Parents were provided with parental counseling. Staff had daily scheduled meetings with both parents and the young person. Families were usually offered family therapy sessions twice a week and some of the patients were offered individual sessions. Supplementary sessions were typically arranged in collaboration with the patient and parents. As a rule these individual sessions were intended to align with the overarching family therapeutic approach and could be viewed as predominantly supportive or motivational sessions, aiming to support the young person’s treatment engagement together with helping them appreciate the greater responsibilities obtained by the parents. During some phases between 2008 and 2014, parents have been offered a parenting course inspired by the skills-based parent program developed at the Maudsley hospital [25], and for the majority of the time period (i.e., between 2008 and 2014) the EDU has provided weekly parent groups facilitated by staff, where the parents themselves were in charge of the content.

At discharge, all patients and families were transferred back to their local mental health services. Although siblings were welcome to take part in the admission, most families arranged for siblings to remain at home during the majority of the hospital admission. Siblings, however, could participate in family therapy sessions and family meals during visits to the EDU. Occasionally, a sibling group has been offered at the EDU led by a senior nurse or clinical psychologist.

### Interview guides and interviews

Interview guides were developed separately for patients, parents and siblings by a group of experienced clinicians led by a senior researcher [IH]. Interview guides were piloted and revised before the final completion. Despite subtle differences, all interview guides were semi-structured and organized into three broad sections to cover perspectives related to the pre- admission phase, admission, and post discharge. Patients and siblings were interviewed individually. Parents were given the opportunity to choose whether they wanted to be interviewed separately or together.

Interviews were administered in 2015. A team of 5 senior clinicians and one advanced psychology student conducted the interviews, with one of the co-authors [IH] administering the majority of interviews (i.e., 14 of 26). The rest of the interviews were administered by the psychology student (i.e., 6 of the sibling interviews), and four specialist nurses (i.e., three specialist nurses conducted one interview each, and one specialist nurse and family therapist administered three interviews). Interviews were transcribed verbatim. Questions included how the participants had experienced the admission, whether they would have preferred any changes based on their experiences, an invitation to give their advice to the treatment providers and peers, together with questions on how they experienced the pre-treatment phase and transitioning back home. The interview guides are available upon request.

### Qualitative analyses

Starting out our analysis was inspired by a multiperspectival interpretative phenomenological analysis (IPA) framework [26]. During the initial process of conducting the analysis according to the steps outlined in multiperspectival IPA, we [JVN & TWH] encountered several dilemmas. In particular, we were concerned whether our data were sufficiently rich enough to utilize an interpretative or hermeneutic approach such as multiperspectival IPA. After thoroughly discussing these important dilemmas we concluded that the raw data, together with the original research question, were most likely better managed while applying a predominantly pragmatic descriptive thematic analysis (TA) approach [27].

Both TA and IPA share much in common. They offer the researcher a set of steps, or a road map, for conducting the analysis, they can both be multiperspectival (i.e., involve participants from different positions as including parents, siblings and patients) and they both aim to generate themes based on the original data. Still the most striking difference, we believe, and this became crucial for our conclusions, is that while IPA has strong historic roots in specifically hermeneutics and phenomenology [26], TA represents a more pragmatic, a-theoretic framework that enables the analytic team to position the analysis in more flexible ways [27]. Critical for the current study was hence the assessment that our data was judged as more suitable for a descriptive TA approach, compared with the more interpretative stance recognizing the IPA framework.

Although unavoidably influenced by our initial analysis, we started over by re-familiarizing ourselves with the raw material while retaining the original multiperspectival approach. The first author [JVN] read and re-read all transcripts together with preliminary coding, applying a more descriptive stance. At the same time, co-author TWH read the complete data set in order assist and collaborate in the evolving process, performing the role as a “critical friend” [28]. Again, we read and coded [chiefly performed by JVN] individual transcripts, one family at the time. We started out with the parents, followed by the index patient, and finally the siblings. Before finalizing the analysis, we scheduled weekly meetings to discuss the iterative process over a 2-month period. This work was done in accordance with the 6 steps outlined in TA [27]. After analyzing the individual interviews case- by- case, we used substantial time to explore whether we could find any thematic development that supported a shared family narrative, that is, we searched for themes potentially shared within the family as a whole, and also for similarities and discrepancies between families.

## Results

A thematic structure of 5 main themes captured 14 subthemes, as outlined below (cf. Table 1 for a brief summary). During the analysis, we did not find evidence of a shared family narrative within the current sample. Rather than constructing “a shared family narrative” or mapping out themes on the “family level,” it was interpreted that the participants’ perspectives were predominantly influenced by both the position in the family (i.e., on an individual or between individuals level) and what we understood as the relationship to the ED. As outlined below, some of the subthemes were related to all family members, and these reflections are captured collectively under the same subtheme, whereas some subthemes represent views from one position alone (i.e., only the parents). All 8 families are represented with data excerpts.

**Table 1:** Results

Main themes	Subthemes
**1: Expectations and evaluation of needs. Entering treatment from different vantage points**	Subtheme 1: “We needed a time-out”: parents appreciating the admission as a much needed restart for the family – *parents* (*N* = 14) Subtheme 2: From opposition to realizing that “something had to happen” – *patients* (*N* = 8) Subtheme 3: The admission arriving as a surprise – *siblings* (*N* = 8)
**2: Interactions with peers during the admission as highly beneficial or problematic**	Subtheme 1: Sharing, learning and recognition of oneself in the other – *parents* (*N* = 14) and *siblings* (*N* = 5) Subtheme 2: Peer interactions as problematic: heightened pressure and symptom contagion – *patients* (*N* = 6) and *parents* (*N* = 2)
**3: Perspectives on staff expertise and the EDU structure**	Subtheme 1: Improved understanding of ED and insight into the young patients challenges – *parents* (*N* = 14) and *siblings* (*N* = 7) Subtheme 2: Strengthening parental authority and re-establishing normalized meal routines – *parents* (*N* = 9) Subtheme 3: Enabling necessary weight gain – *parents* (*N* = 8), *patients* (*N* = 3) and *siblings* (*N* = 4) Subtheme 4: The unintended potential of treatment keeping parents in a bystander position – *parents* (*N* = 5)
**4: Influencing within-family relationships in different ways**	Subtheme 1: Strengthening within family relationships – *siblings* (*N* = 5), *parents* (*N* = 10) and *patients* (*N* = 6) Subtheme 2: The potential of maintaining or increasing fragmentation – *siblings* (*N* = 5) and *parents* (*N* = 4)
**5: Being admitted is at best only half the job: reflections on leaving the EDU**	Subtheme 1: Leaving the EDU while the ED is still on board – *parents* (*N* = 10) and *patients* (*N* = 4) Subtheme 2: Being transferred back to where it did not work out in the first place – *parents* (*N* = 8) and *patients* (*N* = 5) Subtheme 3: For siblings, leaving the EDU meant leaving treatment for good: calling for better sibling involvement – *siblings* (*N* = 8) and *parents* (*N* = 10)

Note: To indicate the robustness of findings, the number of participants sharing views within each subtheme is listed in parenthesis

### Main theme 1: expectations and valuation of needs. Entering treatment from different vantage points

This main theme reflected the perspective that young persons with AN (hereafter abbreviated as YP-AN) and their family members entered treatment from very different vantage points. This variation was predominantly interpreted as contingent on roles and responsibilities at the time of the admission, together with what we determined as the relationship to the ED.

### Subtheme 1: “We needed a time-out”: parents appreciating the admission as a much needed restart for the family

We couldn’t handle the situation at home, we clearly needed help […] it’s obvious. You feel very powerless as a parent when your child stops eating [Anna, *a mother reflecting back on a sensation resonating with most parents prior to the admission. Although engaged in treatment prior to the family-based admission; expressing strong feelings of being disempowered as parents, combined with a growing sense of that “somebody” has to intervene as things were beyond parental control*]

Although some of the parents recalled initial skepticism and ideally wanted to manage the situation at home without intensified treatment efforts, parents entered treatment with an overall high degree of readiness, as most “longed for the admission to finally start.” Generally, parents recalled the pre-admission phase by interpersonal tension and high levels of within-family conflicts. They voiced multiple examples of how the family and individual family members had accommodated to the ED over time. Simultaneously, most parents recollected feeling renewed hope when reflecting on the time prior to the admission; anticipating that this new treatment effort could be helpful, “finally we were going to get help.” Looking back, all parents described “a sense of relief” when the referral to the EDU was accepted. Most parents also recalled they found it important that the EDU was deliberately providing space for the whole family, “as this was a family issue.”

### Subtheme 2: from opposition to realizing that “something had to happen”

Contrary to their parents, all but one of the YP-AN remembered opposing treatment at the time of the admission.

I was kind of… forced… I was really fed up with treatment and did not want to be there [Brenda, 20 years, 14 during the admission, *an extract resonating with most YP-AN at the time of the admission, as they overall recalled low readiness for a new treatment effort*]

Reflecting back from a more distant position, all eight former patients acknowledged that something had to happen at the time of the admission, as they remembered things were not working out at home or even at the treatment facility where they had received therapy.

I guess I thought, “I’m not going to go there”. That it was totally unacceptable. I guess I didn’t imagine that I needed another admission, after [recently] being discharged at the medical ward… […] It was necessary, I see that *now*. That I got help somewhere, so, if it was at [name of unit] or a different place, I don’t know, but it was nevertheless essential that they stopped me from losing further weight… [Molly, 18 years, 15 during the admission*, although her vantage point was characterized by initial opposition, the excerpt showed how her perspectives on being admitted had changed over time*]

One YP-AN reflected contrary views, as she voiced high levels of pre-admission readiness, recalling that she felt extremely exhausted, and “ready for somebody to take over control,” as she recognized that everything pertaining to food and meals was far beyond control. She also remembered thinking that although she really wanted change, she was unable to make the necessary changes alone.

### Subtheme 3: the admission arriving as a surprise

I thought it was very peculiar. Very extraordinary, that my family had to be hospitalized. That my sister, that *she* had any problems? She was very conscientious and was feeling really, very well, I thought […] that she needed help, that there was a problem, that I found very strange [Sister, Catherine, 14 years, 10 during the admission, *reflecting back on the admission arriving as a surprise*]

None of the siblings had previously been involved in family-based treatment for AN. In general, siblings described that the admission came as a big surprise. For the two siblings that did not express this viewpoint, one was apparently well-informed and also very eager to take part in the admission. Resonating with the YP-AN views captured in subtheme 2, some of the siblings recalled feeling oppositional when they learned the admission was family-based and they were expected to participate. For some, the sensation of surprise thus developed into sheer resistance.

I was very negatively inclined. I did not like the fact that we were supposed to be admitted, that I had to stay there. I never stayed there. Me and my little brother were always at home together with either mom or dad […] I remember they asked if I wanted to stay over, but I didn’t want to, I didn’t feel it was right… [Sister, Jenna, 15 years, 12 during the admission, *reflecting back on her immediate reactions when learning she was supposed to be admitted too*]

### Main theme 2: interactions with peers during admission as highly beneficial or problematic

This main theme captured participants’ views on being admitted to a treatment setting in which they had the opportunity to interact with peers. Common for all participants was that the family-based admission represented the first time they were admitted together with other families. The subthemes revealed that peer interactions were viewed as predominantly beneficial (subtheme 1) or problematic (subtheme 2).

### Subtheme 1: sharing, learning and recognition of oneself in the other

I think everybody felt that it was really useful to recognize that others had, in fact, experienced the same, or at least something in the same way. That it wasn’t all about us. I believe that is important for parents too, to know that you’re not alone on this [Father, Paul, *reflecting back on the peer group for parents. Although facilitated by staff, the group focused on issues the parents raised on that particular day*]

Parents and siblings both viewed being admitted together with other families as largely supportive and meaningful. Parents emphasized that having weekly meetings scheduled with other parents was a very supportive experience. They typically recognized that their own within-family struggles, as well as the numerous challenges with the health care system, resonated with others, i.e., “increased feeling of connection,” “we were not the only ones,” “others held similar experiences as ours,” “it was not only us that reacted to such behaviors.” Additionally, some parents remarked that it was often easier to discuss issues with other parents compared to professionals.

For siblings who interacted with other siblings during the admission, the prospect of meeting others was viewed favorably, especially among siblings of the same age with shared interests. Whereas few parents spontaneously interacted with other parents or family members during the admission, siblings reported more frequent encounters.

I think it was pretty nice. Then I understood that it wasn’t only me that had it like that. Somebody else had the same, like me. It felt, I think it was a good thing to be together with somebody else that had similar challenges [Brother, Kenneth, 15.5 years, 12 during the admission; *on the perceived benefit of meeting other siblings during the stay*]

### Subtheme 2: peer interactions as problematic: heightened pressure and symptom contagion

For me, the surroundings were very negative… and I guess I was very susceptible too, and that I think everybody was [YP-AN, Jane, 21 years, 16 during the admission, *on being admitted with peers with severe challenges in a vulnerable phase, a sensation resonating with the majority of the YP-AN when reflecting back on interactions with peers*]

None of the patients shared stories of supportive interactions with fellow patients or other families. Quite the contrary, the YP-AN seemed to strongly feel that being admitted with peers was problematic. They recalled peer interactions frequently led to comparisons and negative competition. Some also acknowledged they, too, likely exerted negative pressure on others. Several of the YP-AN concluded that being admitted with peers with AN is potentially very problematic and should be handled carefully. Overall, parents perceived peer interactions between YP-AN as less problematic, although some did recollect that their child probably learned new and negative symptom behaviors, most likely due to observing and imitating peers during the admission.

### Main theme 3: perspectives on staff expertise and the EDU structure

Both parents and siblings voiced that interacting with, and getting support from, experienced staff together within a structured treatment setting was beneficial for understanding the ED, strengthening parental authority, and re-establishing normalized meal routines. Several also emphasized that the EDU structure and staff expertise were crucial factors enabling weight gain and ED symptom improvement. Finally, this main theme also captured that, although staff expertise and the structure of the EDU were viewed as beneficial overall (especially voiced by parents), some aspects could, in certain instances, be interpreted as non-intentionally maintaining the ED.

### Subtheme 1: improved understanding of the ED and insight into the young patients challenges

That we learned more about the ED. That we could be present… and maybe that mom and dad learned to be more firm when telling my sister that she needed to eat [Brother, Kenneth, *on what he believed was especially valuable for the family; both a better understanding and that the parents were able to manage the meals more efficiently*]

Most of the parents, and some of the siblings, recalled benefiting from the staff’s expertise, which improved their general knowledge of EDs, as well as their specific understanding of the unique challenges facing the YP-AN. Several of the parents, and siblings, implied that greater knowledge and awareness enhanced empathy, i.e. “when we were able to see how difficult it was, we could understand better how it really was for her.” Despite having undergone extensive prior treatment, including previous hospitalizations, quite a few parents and siblings emphasized this was the first time they truly had the opportunity to learn about the ED. The educational program for parents was viewed as particularly beneficial in improving knowledge about the ED, and how the ED challenged the parental role.

Attending the parenting courses was very helpful. Then you got something concrete to relate things to, and that helped, I think [Mother, Caroline, *recalling how learning more about ED and being introduced to how the ED typically challenges parenting was useful for her*]

### Subtheme 2: strengthening parental authority and re-establishing normalized meal routines

To learn to be calmer during meals. I think we were able to manage the meals more peacefully while on the unit, compared with previously, then there was no such thing as a calm meal! And we got rid of weighing the food [Mother, Ruth, *reflecting on the potential benefit of breaking patterns while being socialized into a meal structure compatible with a more normalized family life, and the prospect of un-learning of non-supportive behaviors*]

Parents highlighted several aspects of the EDU structure as particularly beneficial for breaking patterns and in re-establishing normalized meal routines.

That I felt *so* secure, that the [meal] structure was so firm […] that was the first thing I was very satisfied with, that somebody, like, took the responsibility from us, so we could have some real help, since we didn’t manage it [at home] [Mother, Sarah, *on the potential benefit of parents being able to lean on a structure administered by the professionals*]

Following Sarah’s excerpt, the father continued to describe how the established routines and structure at the EDU aided in re-installing parental authority, which had more or less vanished under the pressures of the ED.

It was a very welcome feeling of not standing alone with everything […] We were, in a way, defeated as parents, and how should I put it? Ehm, we had no authority, no influence; we were no longer defined by our daughter as caregivers in relation to food. I think our daughter didn’t perceive that we had anything reasonable to say concerning food, because she was so convinced she was right. So, to come here and get support for the parenting, that felt very meaningful [Father, Peter, *on the EDUs potential of reinstalling and supporting parental authority*]

Sarah (wife) later joined in and summed it up:

We regained a belief in our ability to function as parents […] we recovered self-confidence and a belief in that we can be parents and authority figures for our daughter [Mother, Sarah]

### Subtheme 3: enabling necessary weight gain

Although some of the YP-AN retrospectively acknowledged the necessity of weight gain to recover, parents and some siblings strongly emphasized the benefits of the admission in facilitating improvement on physical parameters. Weight gain and medical outcomes were predominantly ascribed to staff expertise, and enabled by the structure of the EDU, more than fueled by increased parental self-efficacy. Yet weight gain and related improvements were not uniformly perceived as linked with improved psychological well-being, as reflected in Caroline’s quote below:

To gain weight, you talked a lot about that, that it was supposed to help, and then you were supposed to get a clearer mind. We’ve witnessed quite the contrary with her [Mother, Caroline*, referring to how she remembered that although emphasizing the inevitable necessity of weight gain; how difficult it was when her daughter Jane actually gained weight, and that psychological symptoms did not immediately recede as she felt she had been told over and over again*]

### Subtheme 4: the unintended potential of treatment keeping parents in a bystander position

We didn’t perceive ourselves as so important [during the admission]. It was more that our son was prioritized. That was most important [Father, Steven]

Although most parents voiced an initial need to step back and “let the experts take care of an unmanageable situation,” the majority retrospectively perceived that treatment strengthened their role and position as caregivers (i.e., as reflected by the majority in subtheme 2 above). Still, we interpreted some parental views as acknowledging the potential of the treatment to maintain them in a bystander or sidelined position. For some, it was as if treatment failed to co-construct a collaborative relationship that strengthened their parental authority and relational agency.

Paul [Father]: I think, for my part, that it was reassuring that somebody could help my daughter, like, “Now we know she gets what she needs”, “Now she’s going to get better”, that I felt was very reassuring […] still I felt that it was difficult. I didn’t feel that *I* took part. I don’t know if this was because I opted out or not, but I don’t think so, it was like, you were supposed to join in and take part, still you were on the sideline […] It was like, the one who controlled everything and had the direction, it was that therapist, or the one being present at that moment [that were in charge] and I was in a way set aside, as I felt it…

Inger [Interviewer]: The therapist took over?

Paul [Father]: Yes, it was like that in a way, and further, I noticed on my daughter too […] like, she really needed to hear it from somebody [else] what she should do too, and it became much easier for her to listen to somebody else, of course, that knows this*.*

As we read this excerpt, together with other parental excerpts that touched upon Paul’s perspectives, we recognized the potential of the YP-AN becoming dependent upon staff instructions and / or authority. This development could ultimately become a hindrance in aiding parental efficacy, and reinforce the idea that staff members are the “true” experts, thereby maintaining parents in a bystander position.

### Main theme 4: influencing within-family relationships

This main theme captured contrasting views on how the admission was perceived as supportive and strengthening within-family relationships, while others viewed the admission as maintaining or increasing fragmentation.

### Subtheme 1: strengthening within-family relationships

Parents and siblings shared a range of views relating to reduced relational distance, i.e., “we came closer,” improved collaboration,” “we managed to collaborate better.” Several voiced enhanced within-family understanding of each other and the ED, i.e. “by being together we learned together and understood better,” and reduced within-family conflicts, i.e., “things became calmer.”

Sarah [Mother]: We felt we came closer to each other, that our collaboration improved, or…

Peter [Father]: Mhm… we experienced that as a family, too. All these conversations we had, and the groups and, yes, both the individual family sessions and couple sessions we had, and these group meetings with the other parents. Everything helped us to sort things out between us… so our relationship and to our daughter… I think it became a closer relationship [*Both parents reflecting on noticing improved collaboration and strengthened relationships*]

Although few of the YP-AN emphasized that having been admitted was aiding them directly (i.e., as personally perceived as supportive at the time), some reflected as Jane below, that although the admission paralleled an extremely difficult time period, looking back she had come to appreciate that the admission was of benefit for her parents, the family, and in strengthening relationships:

When I think back, I do believe it is the worst thing I’ve ever experienced [reflecting back on the time of the admission] [still] I did observe, there, that my parents seemed a bit happier, calmer. At home, I felt it was like, police and thief, and our relationship was suffering when we were at home [prior to the admission], and I felt it was strengthened when we were there. They became more my supporters […] I would say it was of benefit for my family… [Jane, 21 years, 16 during the admission, *recalling that although the admission represented the worst of memories, it was beneficial for the family*]

### Subtheme 2: the potential of maintaining or increasing fragmentation

Although we assume that “living with the ED” had contributed to an increased sense of separateness for the afflicted families, some of the participants voiced concerns that the organization of the admission might represent a further division for some families, i.e., “as we did not stay there together, we became even more divided”.

I feel in a way that we came closer to each other, but also that we in ways became divided. Mom was with my sister all the time [at the EDU], and then it was us three [at home]. We too came a bit closer, still it was a bit divided [Sister, Angie, 15 years, 12 during the admission, *reflecting on the feeling of both getting closer with some family-members, and at the same time; a sensation of being divided*]

This sense of disconnectedness was particularly echoed in some of the siblings’ accounts. In particular, some of the youngest siblings found it challenging to spend less time with the parent who was frequently at the EDU; typically this was their mother. On the other hand, some siblings voiced the benefits of an improved relationship with their father as a consequence. This feeling of disconnectedness also resonated with some of the parents, who emphasized that if they could do “one thing over again,” it would be to be admitted earlier to the specialized EDU, and to stay together as an entire family. These parents now believed that “they” as parents and “we” as the family would have benefited more from an earlier admission that included all family members.

### Main theme 5: being admitted is at best only half the job: reflections on leaving the EDU

This main theme captured realizations that discharge did not represent the end of living with an ED, or even signify the end of treatment, as some of the family members may have anticipated or hoped for initially when admitted. Although many viewed several aspects of hospitalization as beneficial, both for themselves and their family, it was clear that discharge from the EDU represented at best only half the job.

### Subtheme 1: leaving the EDU while the ED is still on board

It was very final, at least for us, when we were discharged, it was like “goodbye” and that’s it. We never made any calls and I guess there were no openings either? We never heard that we could, and we didn’t do it anyhow. I guess we probably could have done it, and maybe have the chance to have a conversation with somebody, but we felt it was very final, that we were not supposed to make any calls [to the EDU] and I guess it often feels like this, that it is a bit abrupt after such a long admission [Mother, Linda, *reflecting back on discharge*]

The majority of the participants remembered the immediate phase following discharge as very difficult. The ED was still present and exerted a great influence on the YP-AN and daily life as a family. Despite practice managing the recommended meal structure at the EDU during planned leaves, several parents acknowledged a prolonged admission or additional follow-up at the EDU as potentially beneficial after discharge. Some parents suggested that a scheduled brief “booster” re-admission would be beneficial, without having to undergo a full relapse to gain re-admission at the EDU or inpatient treatment elsewhere. Although discharge was known in advance and planned to a certain extent, several of the parents still perceived discharge as occurring suddenly and implied that it was not properly planned.

Even some of the YP-AN who initially resisted hospitalization felt the admission ended abruptly with insufficient planning and predictability. Some even reflected that a longer admission would have been beneficial, as they realized they had remaining ground to cover.

When I was admitted, at the time I didn’t eat by myself [nasogastric tube] … Nor did I start with serving myself, and [thus] did never practice that, so, that I think was something we could have worked on… [Diana, 20 years, 17 during the admission, *on the potential benefit of having progressed further before being discharged*]

### Subtheme 2: being transferred back to where it did not work out in the first place

I didn’t feel they had sufficient expertise; they didn’t follow up appropriately [Father, Anthony, *on the decision of not going back to the local outpatient clinic after discharge*]

Most parents voiced concerns related to a treatment impasse at the local outpatient clinic prior to the admission, and found it difficult to accept a referral back to a treatment setting “where it did not work out in the first place.” The majority had lost confidence in the local outpatient clinic and doubted the treatment team could provide assistance following admission to the family-based inpatient program. Skepticism was probably fueled by previous encounters and likely reinforced by receiving highly specialized treatment at the EDU. Similarly, several of the YP-AN also reflected on the paradox of being referred back to the same treatment setting where treatment had previously failed.

I was sent back to the outpatient clinic where I had been prior to the admission and that did not work out at all. And the fact that I was sent back to that place, that was kind of… yes, it did not work out to say it bluntly. So, I’m having a hard time figuring out that one, why it was like that […] And I met a person at the outpatient clinic that didn’t know much, and that was very frustrating and contributed to the ED growing and gained more space again [Molly, 18 years, 15 during the admission, *on finding it difficult to accept that she had to go back to where it did not work, while implying how crucial expertise can be to prevent things getting worse*]

One solution for some families involved seeking treatment at a private practice instead of returning to the local outpatient clinic. Although initiated by parents, the decision resonated with the YP-AN’s skepticism in returning to treatment at the local outpatient clinic.

We didn’t go back to the outpatient clinic, because we couldn’t see that there was any therapist there that understood anything of this, and I have to say that we were very lucky to get in touch with a private practitioner, so we started there [Mother, Caroline, *on the difficulties with trusting the local outpatient clinic for further follow up post discharge, and recalling how all in all satisfied she was with finding an experienced private practitioner for her daughter and their family*]

### Subtheme 3: for siblings, leaving the EDU meant leaving treatment for good: calling for better sibling involvement

Siblings also recalled continued hardships for the families following discharge. None of the siblings received additional involvement in treatment post-discharge. Upon reflection, parents and siblings called for a greater focus on siblings during the admission, as “siblings are an equally important part of the family,” including siblings beyond chance meetings and an occasional session with a therapist.

## Discussion

The current study contributes novel knowledge regarding user experiences which can supplement emerging research on adapting core aspects of evidence-based outpatient FBT into higher levels of care [6, 7, 16, 17]. Findings revealed five main themes capturing 14 subthemes (cf. Table 1 for brief summary). No evidence was found of any shared post-treatment family narrative. Participants demonstrated considerable diversity in viewpoints, which was interpreted as being contingent upon their role in the family, responsibilities and relationship to the ED. Without prescribing definitive answers, we believe the results have several implications for treatment providers working within a family-based inpatient treatment approach.

Main theme 1: *Expectations and evaluation of needs. Entering treatment from different vantage points*. This main theme is a useful reminder of the importance of recognizing and valuing the individual needs of families, and refraining from making immediate generalizations of YP-AN and their family members. Families are inevitably constituted by individuals that think, feel and behave differently, even while navigating the apparent “same” social phenomena such as hospitalization. During the pre-admission phase, we believe it is critical to allow sufficient time to explore central issues together with the YP-AN, their family, and the referral system. The findings suggest that different levels of readiness for change, knowledge of the ED as well as preparedness for the admission, in addition to varied expectations and needs are important to explore in-depth prior to an admission. Therefore, we strongly recommend that pre-admission sessions move beyond simply sharing information about the treatment program. The treatment team should enable sufficient time to transparently explore the mutual expectations of family members and treatment providers, investigate previous treatment experiences in-depth, and begin negotiating roles and responsibilities aligning with the overarching family-based treatment approach. Theme 1 also suggests the potential of providing YP-AN and their family members more structured or planned interventions prior to the admission. Without prescribing specific types of interventions, we would recommend the consideration of motivational enhancement sessions for the YP-AN [29, 30] in addition to a brief education program for parents aligning with the skills and content espoused by a family-based approach [25, 31]. It is feasible that an investment in greater resources prior to the admission may optimize the starting point and help the admission become more efficient. Lastly, the first theme emphasizes the importance of enhancing the focus on sibling involvement prior to the admission. Parents should not be left alone in determining how siblings should be informed and / or involved, as sibling involvement should naturally constitute a part of pre-treatment planning for a family-based admission for adolescent AN.

Main theme 2: *Interactions with peers during the admission as highly beneficial or problematic*. The finding that parents valued the mutual support and sharing of experiences with other families is consistent with prior studies of parental peer support and treatment satisfaction in multi-family group therapy [1]. Similarly, the difficulties in navigating peer relationships experienced by YP-AN during admission have also been reported in previous studies [19, 32, 33]. Siblings’ perspectives indicated the benefit of engaging with other siblings, highlighting the importance of enhanced sibling interactions during admissions. Overall, the second main theme suggests the importance of strengthening multi-family work during admissions [34]. Inspired by these findings, we recommend that treatment providers carefully review how peer interactions are enabled and managed during admissions, and to evaluate how the inpatient context can be further optimized to utilize the rich knowledge base embedded in the family members’ lived experiences [1, 35]. Specifically, results remind treatment providers to carefully identify and counter negative peer dynamics between the YP-AN during admissions, and to create opportunities to facilitate peer support. The latter is a potential direction of further investigation in collaboration with YP-AN who have prior inpatient treatment experience.

Main theme 3: *Perspectives on staff expertise and the EDU structure*. The majority of parents viewed the EDU structure and staff expertise as aiding their perceived parental self-efficacy, which is one of the proposed mechanisms of change in family-based treatments [6]. It is encouraging that most parents reported observable behavior change or symptom improvement, not simply treatment satisfaction. Such improvements tended to generally be ascribed to the opportunity to interact with knowledgeable staff and being supported by the EDU structures. Although encouraging, we believe the EDU needs to continue focusing on enabling parental empowerment during admissions [6, 11]. Importantly, the current findings suggest that perceived enhanced parental self-efficacy was not universally experienced. Similar to outpatient FBT [8], inpatient admission is not a panacea, and there is no “one way” to empower all parents. As parents and families enter treatment with unique vulnerabilities, experiences and needs, the therapeutic task of empowering parents must be continually negotiated and tailored to the individual parent’s needs and vantage point.

Main theme 4: *Influencing within-family relationships in different ways*. Findings indicated that treatment was generally perceived to strengthen within-family relationships. This is a reassuring finding, as preventing fissures in relationships and strengthening collaboration within families comprise the core tenants of family-based treatment. Findings are also in accordance with an Australian study of an intensive 2-week family admission program [18]. Although findings generally aligned with the rationale for offering a family-based inpatient program, findings also question the family-based foundation of the program design, as none of the participating families stayed for the entire length of the admission. Thus, it is reasonable to question how family-based the program “really” is, when important members of the family system were rarely represented at the EDU, and thus not actively engaged in treatment. Involving the “whole” family is usually advocated in the literature, as this constitutes a pillar when providing treatment. Still, the prominent stance of “including the family” is often far removed from the day-to-day realities. This seems to resonate with research showing that clinicians regularly fail to sufficiently involve family members even when providing standard FBT, a treatment model that explicitly aims to include the family [36, 37]. Admitting a family for a prolonged time period is obviously demanding on resources and represents a highly complex treatment situation for which clear evidence to guide treatment providers is scarce. Research is sorely needed to understand how to best optimize the inpatient setting and to investigate whether engaging the whole family to a greater extent during hospitalizations can improve outcome and facilitate successful transitions after discharge.

Main theme 5: *Being admitted is at best only half the job: reflections on leaving the EDU*. In accordance with previous literature [12, 38], transitioning between services represented a vulnerable phase for our families. In general, findings suggested that clinicians carefully plan discharge with the family, and maintain a collaborative relationship with the referral system during the admission. As suggested by our findings, we believe that planning for discharge, and the vulnerable phase after the admission, needs to be properly addressed early during the admission. This includes exploring the expectations of family members, as well as the treatment providers responsible for referral and aftercare, regarding the goals of admission. This effort ensures expectations and goals are transparent, and can help orient everyone involved about the “reality” of the admission being a temporary part of the journey toward recovery [4]. All YP-AN, by definition, will need further specialized care after participation in the family-based admission, and therefore, a plan for the follow-up phase should ideally be decided upon prior to the admission and negotiated based on treatment progression. Collaboration with the referral system should be given more attention prior to the admission, and during treatment, in order to minimize the likelihood of families perceiving discharge as abrupt and poorly planned.

## Strengths and limitations

Investigating user perspectives from three different positions (patient, parents, and siblings) is viewed as a strength. Throughout the analysis, we maintained a focus on the family. In our view, the experience of families is perhaps paradoxically lacking in many qualitative studies of family-based interventions, which often focus on the single views of the patient, parents, or siblings. An obvious limitation is the retrospective nature of the study. Unquestionably, the time elapsed between discharge and follow-up interviews may influence participants’ recollections. Still, time has also enabled participants to reflect from a potentially more mature, self-reflexive, and thus, less emotionally-laden position, compared to being interviewed shortly after discharge. Another limitation is the sampling strategy. As few intact families were available in the dataset (*N* = 8), results cannot be generalized broadly, and different families may have provided difference responses. Thus, the analysis does not claim to provide a narrative on how family members generally experience family-based inpatient treatment. In addition, the specialized EDU treatment setting which offered treatment comprises a specific context not necessarily generalizable to other regions and countries. Still, we believe the findings, in addition to clinical implications derived, offer valuable insight and are relevant for treatment providers aiming to optimize family-based treatment at higher levels of care. Another limitation is that several interviewers with varying levels of interview skills took part in conducting the interviews. This may have affected the richness of the data. We also question whether the retrospective interview data, as in the current study, provides the best data source to inform further treatment development, which is the overarching aim for our qualitative research projects. Future research should aim to generate more detailed descriptions to guide the development of family-based treatment for adolescent AN at higher levels of care. We suggest improving the system for administering interviews (e.g., to administer interviews both during treatment and soon after discharge), together with ethnographic fieldwork in order to study practice as it unfolds in real time. Lastly, a potential limitation worth mentioning is that the patient and sibling transcripts have been utilized in our previous research, although with a different research purpose. This can have influenced both analysis and findings in the current study.

## Conclusions

Our study offers insight into how former inpatients and their family members experienced an inpatient treatment program designed to align treatment with the central elements of an outpatient family-based treatment approach for adolescent anorexia nervosa. Overall, the findings support emerging research underlining the necessity of strengthening the family-based treatment approach within intensified treatment settings. Moreover, the results emphasized the need for more knowledge on how to optimize inpatient treatment as well as the importance of providing smooth transitions between care settings.

## Data Availability

The dataset collected and analyzed during the current study are not publicly available as this could compromise participant privacy. The corresponding author can be contacted on reasonable request with questions considering the dataset.

## Abbreviations

AN: Anorexia nervosa
FBT: Family-based treatment
EDU: Eating disorder unit
YP-AN: Young person with anorexia nervosa
TA: Thematic analysis
IPA: Interpretative phenomenological analysis
